# Smartphone internet addiction among hong kong young adults: The role of gender and depression

**DOI:** 10.1192/j.eurpsy.2021.562

**Published:** 2021-08-13

**Authors:** D.L. Chan, C.S. Wong, C.L. Hui, S.K. Chan, E.H. Lee

**Affiliations:** 1 Psychiatry, The University of Hong Kong, Hong Kong, Hong Kong PRC; 2 Department Of Psychiatry, The University of Hong Kong, Hong Kong, Hong Kong PRC; 3 Department Of Psychiatry, The University of Hong Kong, HK, Hong Kong PRC

**Keywords:** Internet addiction, smartphone, community, Depression

## Abstract

**Introduction:**

Growing evidence studying pathological online behaviour has shown an increasing rate of internet addictions in younger populations across the globe.

**Objectives:**

The current study aims to investigate the prevalence of smartphone internet addiction of youths in Hong Kong, and its associations with gender and depression.

**Methods:**

A total of 1,164 participants’ preliminary data were extracted from the Hong Kong Youth Epidemiological Study of Mental Health, a territory-wide, household-based study of mental health in youths aged between 15-24. Internet usage behaviors, socio-demographic and psychosocial characteristics of the participants were assessed. The Chen Internet Addiction Scale was modified to measure smartphone internet addiction (SIA). Symptoms of depression were assessed using the Patient Health Questionnaire. Mann-Whitney U tests were used to examine (i) SIA across gender and (ii) depressive symptoms between high and no to low SIA groups. Linear regression model was used to evaluate the association between SIA and depression.

**Results:**

The prevalence of smartphone internet addiction was 27.8% using the cut-off scores of 67/68. Women had higher SIA scores than men (U=144239.50, p=0.001). Participants with high SIA were associated with a higher severity in depression than those with no-to-low SIA (U=89187.00, p<0.001). Regression analysis revealed a significant positive correlation between depression and SIA after adjusting for confounding factors (B=0.099, t=9.138, p<0.001).

**Conclusions:**

Our findings suggest a gender difference on online behaviour using smartphones. Further investigations are needed on whether SIA may exacerbate severity of common mental disorders.

